# Draft Genome Sequences of Gammaproteobacterial Methanotrophs Isolated from Lake Washington Sediment

**DOI:** 10.1128/genomeA.00103-15

**Published:** 2015-03-12

**Authors:** Marina G. Kalyuzhnaya, Andrew E. Lamb, Tami L. McTaggart, Igor Y. Oshkin, Nicole Shapiro, Tanja Woyke, Ludmila Chistoserdova

**Affiliations:** a Department of Microbiology, University of Washington, Seattle, Washington, USA; b Department of Chemical Engineering, University of Washington, Seattle, Washington, USA; c DOE Joint Genome Institute, Walnut Creek, California, USA

## Abstract

The genomes of Methylosarcina lacus LW14^T^ (=ATCC BAA-1047^T^ = JCM 13284^T^), Methylobacter sp. strain 21/22, Methylobacter sp. strain 31/32, Methylomonas sp. strain LW13, Methylomonas sp. strain MK1, and Methylomonas sp. strain 11b were sequenced and are reported here. All the strains are obligately methanotrophic bacteria isolated from the sediment of Lake Washington.

## GENOME ANNOUNCEMENT

Lake Washington sediment has served for decades as a model system for environmental studies on aerobic methane oxidation (1–8). A great deal of information has been accumulated regarding methane consumption rates (1, 3) and the composition of microbial communities that consume methane (4–8). A number of methanotroph species have been isolated in pure culture (3). Here, we announce the draft genome sequences of six obligately methanotrophic bacteria belonging to the family *Methylococcaceae* (Table 1).

**TABLE 1:** Strains described, accession numbers, and general genome statistics

Strain	Sample collection date	NCBI accession no.	Sequencing platform (genome coverage [×])	No. of scaffolds (no. of contigs)	G+C content (%)	Size (Mb)	Gene count^*a*^	pMMO/sMMO^*b*^	Nif^*c*^	Hyd^*d*^
Methylobacter sp. 21/22	2011	JMLA00000000	PacBio (79)	4 (4)	49.5	4.7	4,239	2/0	+	+
Methylobacter sp. 31/32	2011	JPOH00000000	PacBio (104)	2 (2)	49.2	5.0	4,669	2/0	+	+
Methylomonas sp. 11b	2011	AZXK00000000	Illumina (755)	1 (2)	51.4	5.4	5,086	3/1	+	+
Methylomonas sp. MK1	2011	AQOV00000000	PacBio/Illumina (79)	5 (5)	51.5	5.2	4,851	3/0	+	+
Methylomonas sp. LW13	1999	JNLB00000000	Illumina (300)	42 (42)	51.8	5.2	4,806	2/1	+	+
Methylosarcina lacus LW14^T^	1999	AZUN00000000	Illumina (912)	1 (1)	54.7	4.4	4,047	1/0	−	−

a Number of genomic objects (coding sequences [CDSs], fragmented CDSs [fCDS], rRNA, tRNA, miscellaneous RNA [miscRNA]).

b Number of gene clusters encoding particulate methane monooxygenase (pMMO) or soluble methane monooxygenase (sMMO).

c Nitrogenase gene cluster. +, present; −, absent.

d Hydrogenase (NiFe) gene cluster.

Strains Methylomonas sp. LW13 and Methylosarcina lacus LW14^T^ were isolated in 1999 (3), and strain LW14^T^ has been formally described (9). Strains Methylomonas sp. 11b, Methylomonas sp. MK1, Methylobacter sp. 21/22, and Methylobacter sp. 31/32 were isolated from a sample collected in 2011 (7, 8). The Methylobacter strains are psychrophilic and do not grow at temperatures of >24°C, while the remaining strains grow well at 30°C (3, 9). DNA preparations were obtained using the phenol-chloroform method (9). The draft genome sequences were generated at the Department of Energy (DOE) Joint Genome Institute (JGI), Walnut Creek, CA, USA, using the Illumina platform (10) or Pacific Biosciences (PacBio) technology (Table 1). The raw reads were assembled using HGAP (version 2.1.1; PacBio data) (11) or AllPaths, version 39750 (12) and/or Velvet, version 1.1.05 (Illumina data). All general aspects of library construction and sequencing performed at the JGI can be found at http://www.jgi.doe.gov. Genome annotation was performed using Prodigal (13), followed by a round of manual curation using GenePRIMP (14) for the genomes in <20 scaffolds. The predicted coding sequences were translated and used to search the National Center for Biotechnology Information (NCBI) nonredundant database and the UniProt, TIGRFam, Pfam, KEGG, COG, and InterPro databases. Additional gene prediction analysis was performed within the Integrated Microbial Genomes (IMG) platform (15). The genome statistics are shown in Table 1.

As typical obligate methanotrophs, all the strains encode particulate methane monooxygenase for methane oxidation. In addition, soluble methane monooxygenase gene clusters were identified in the genomes of Methylomonas sp. strain 11b and Methylomonas sp. strain LW13. The complete gene inventories for the ribulose monophosphate pathway (both KDPG [2-keto-3-deoxy-6-phosphogluconate] and FBA [fructose 1,6-bisphosphate aldolase] variants [16]) are present in all genomes. All three Methylomonas strains, as well as M. lacus LW14^T^, also possess complete sets of genes for the serine cycle, while in the Methylobacter species, the genes coding for one enzyme, phosphoenolpyruvate carboxylase, are not identifiable. None of the strains encode ribulose-1,5-bisphosphate carboxylase/oxygenase or its homologues. With the exception of M. lacus LW14^T^, all the organisms encode functions for nitrogen fixation and hydrogen production/utilization. Respiratory nitrate-nitrite reductases are encoded only in the Methylobacter sp. genomes, while nitrite/nitrous oxide reductases are encoded only in the Methylomonas sp. and M. lacus LW14^T^ genomes. With the availability of their genomic sequences, these diverse *Methylococcaceae* isolates present prospective models for studying methanotrophy in freshwater lake sediments.

### Nucleotide sequence accession numbers.

The genome sequences have been deposited in GenBank under the accession numbers listed in Table 1.
